# miR-150 inhibits terminal erythroid proliferation and differentiation

**DOI:** 10.18632/oncotarget.5824

**Published:** 2015-11-03

**Authors:** Zhiwei Sun, Ye Wang, Xu Han, Xielan Zhao, Yuanliang Peng, Yusheng Li, Minyuan Peng, Jianhui Song, Kunlu Wu, Shumin Sun, Weihua Zhou, Biwei Qi, Chufan Zhou, Huiyong Chen, Xiuli An, Jing Liu

**Affiliations:** ^1^ The State Key Laboratory of Medical Genetics & School of Life Sciences, Central South University, Changsha 410078, China; ^2^ Xiangya Hospital, Central South University, Changsha 410008, China; ^3^ College of Life Sciences, Zhengzhou University, Zhengzhou 450001, China; ^4^ Laboratory of Membrane Biology, New York Blood Center, New York, NY 10065, USA

**Keywords:** miR-150, terminal erythropoiesis, 4.1R, erythroid proliferation, transcriptional profiling

## Abstract

MicroRNAs (miRNAs), a class of small non-coding linear RNAs, have been shown to play a crucial role in erythropoiesis. To evaluate the indispensable role of constant suppression of miR-150 during terminal erythropoiesis, we performed miR-150 gain- and loss-of-function experiments on hemin-induced K562 cells and EPO-induced human CD34^+^ cells. We found that forced expression of miR-150 suppresses commitment of hemoglobinization and CD235a labeling in both cell types. Erythroid proliferation is also inhibited via inducing apoptosis and blocking the cell cycle when miR-150 is overexpressed. In contrast, miR-150 inhibition promotes terminal erythropoiesis. 4.1 R gene is a new target of miR-150 during terminal erythropoiesis, and its abundance ensures the mechanical stability and deformability of the membrane. However, knockdown of 4.1 R did not affect terminal erythropoiesis. Transcriptional profiling identified more molecules involved in terminal erythroid dysregulation derived from miR-150 overexpression. These results shed light on the role of miR-150 during human terminal erythropoiesis. This is the first report highlighting the relationship between miRNA and membrane protein and enhancing our understanding of how miRNA works in the hematopoietic system.

## INTRODUCTION

Erythropoiesis is a hematopoietic process that is tightly regulated via cell lineage specification, proliferation, and differentiation [1–4]. During erythropoiesis, cells undergo several phases, including burst-forming units (BFU-E), colony-forming units (CFU-E), pro-erythroblasts, basophilic erythroblasts, polychromatic erythroblasts, orthochromatic erythroblasts, reticulocytes, and mature red blood cells. The last 6 stages represent terminal erythroid differentiation, characterized by serial cellular changes occurring in membrane-cytoskeleton matrix assembly, cell size, cytoplasmic composition of nucleotide acid and hemoglobin, nuclear-cytoplasmic ratio, and even enucleation [3, 5–7]. Multiple levels of gene expression control and comprehensive gene coordination are required to ensure the proper generation of mature and functional red cells through erythropoiesis [8–15]. Any disruption of erythroid regulatory networks will lead to disease, so identifying and characterizing novel modulators will provide crucial new opportunities for managing erythroid disorders and for the *ex-vivo* generation of red blood cells.

MicroRNAs (miRNAs), a class of small, non-coding linear RNAs, have been demonstrated to play important roles in posttranscriptional gene regulation in both health and disease, such as cell proliferation and differentiation, ontogenesis and tumorigenesis [16–19]. It has been shown that miRNAs also play a crucial role in erythropoiesis. Overexpression of miR-223 blocked the commitment of erythroid progenitors [20], whereas up-regulating miR-210 promoted erythropoiesis [21]. Deficiency or attenuation of miR-144 and miR-451 has been shown to impair late erythroid maturation, which then leads to splenomegaly, erythroid hyperplasia and mild anemia [22–24]. The functions of miRNAs are target dependent. It has been reported that miR-221/222, miR-24, miR-191 and microRNA-146b-5p modulate erythropoiesis through targeting c-kit, Alk4, Riok3 and Mxi1, and PDGFRA, Klfd, respectively [25–28].

Functional miR-150 is 22 nucleotides long, and its gene is located at chromosome 19q13.33. miR-150 was found to be stimulated and drive megakaryocyte-erythrocyte progenitor differentiation toward megakaryocytes at the expense of erythroid cells in the context of thrombopoietin induction [29]. The targeted regulation of the transcription factor c-Myb (MYB) by miR-150 has been studied in lymphoid, myeloid and megakaryocytic lineages [29–33]. However, it remains unknown whether miR-150 suppression is indispensable for terminal erythroid regulation and how it functions. In this study, we focus on terminal erythroid differentiation, using miR-150 gain- and loss-of-function experiments to elucidate the related mechanisms. We confirm that forced miR-150 expression causes inhibition of erythroid cell differentiation and proliferation, and that miR-150 sustained depression favors terminal erythropoiesis. We identify the gene coding red blood cell membrane protein 4.1R as a new specific target of miR-150 in the late stages of terminal erythropoiesis. This is the first report to highlight the relationship between miRNA and red blood cell membrane protein, providing new insight into terminal erythroid maturation.

## RESULTS

### miR-150 expression decreases during terminal erythropoiesis

**Figure 1:**
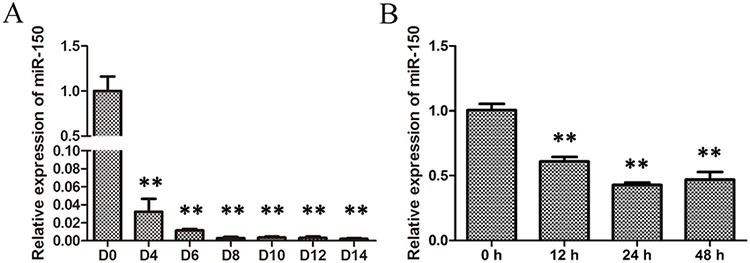
Expression of miR-150 during erythroid differentiation **A.** qRT-PCR analysis of miR-150 during EPO-induced erythroid differentiation of CD34^+^ cells on culture days 0, 4, 6, 8, 10, 12 and 14; **B.** qRT-PCR analysis of miR-150 during hemin-induced erythroid differentiation of K562 cells at 0, 12, 24 and 48 h after induction. U6 snRNA was used as a housekeeping gene, and the results were normalized to the starting time point in each experiments. All data represent mean ±SD (*n* = 3). **P ≤ 0.01 compared with the starting time point.

To explore a potential regulatory role of miR-150 during erythropoiesis, human CD34^+^ hematopoietic progenitor cells were first purified from umbilical cord blood and then induced into erythroid lineage differentiation according to the procedure described in the methods section. The induced cells were collected on culture days 0, 4, 6, 8, 10, 12, and 14, and then total RNA was extracted to assess the levels of miR-150. Data obtained from qRT-PCR demonstrate that miR-150 levels dramatically decreased since D 0 and remained much low after D 8 throughout the subsequent time points of erythroid terminal differentiation (Figure 1A), indicating that miR-150 may have a negative role during erythroblast differentiation. The human erythroleukemia cell line K562 has the potential to be induced into erythroid cells with presence of 50 μM hemin and is often used as a model of erythroid differentiation *in vitro* [28, 34–35]. Total RNA was extracted at 0, 24, 36, and 48 hours after hemin induction, and qRT-PCR was carried out to assess the relative expression of miR-150. Similarly, the relative expression of miR-150 declined significantly after the 24 h time point (Figure 1B).

### miR-150 inhibits hemin-induced erythroid differentiation in K562 cells

**Figure 2:**
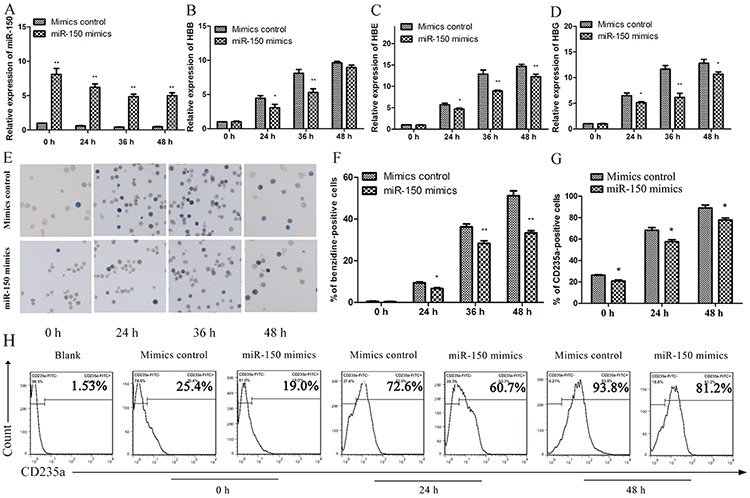
Overexpression of miR-150 inhibits hemin-induced erythroid differentiation of K562 cells K562 cells transiently transfected with miR-150 mimics or controls for the mimics were induced by hemin and evaluated at 0, 24, 36 or 48 h after induction. Real-time qRT-PCR analysis of miR-150 **A.** HBB **B.** HBE **C.** and HBG **D.** Note that U6 or GAPDH was used as the control gene and that results were normalized to the control group at 0 h. Representative images **E.** and statistics **F.** of benzidine staining of hemoglobin-containing cells. Statistics **G.** and representative images **H.** of flow cytometry analysis of CD235a-positive cells. All data represent mean ± SD (*n* = 3). *P < 0.05 and **P < 0.01 for each paired condition with and without miR-150 overexpression.

Based on the above observations, we proceeded to establish miR-150 as a negative regulator during erythropoiesis. K562 cells were transfected with miR-150 mimics, and those cells exhibited robust expression of miR-150 compared to cells transfected with the mimic controls throughout the induction process (Figure 2A). β globin (HBB), γ globin (HBG), ε globin (HBE) and CD235a (GPA, also known as GlyA) were used as erythroid-lineage markers to assess erythroid differentiation after hemin induction. Enforced miR-150 expression produced relatively suppressed erythroid-lineage marker expression levels in each paired condition (Figure 2C and 2D). We also performed benzidine staining to detect the fraction of hemoglobin-containing cells, an indicator of differentiated erythrocytes (blue cells). Compared to the control cells, miR-150 overexpression can reduce the occurrence of hemoglobin-containing erythroid cells: 0.53 ± 0.82% vs. 0.33 ± 0.12% (0 h), 9.40 ± 0.63% vs. 6.63 ± 0.61% (24 h), 36.17 ± 2.40% vs. 28.27 ± 2.29% (36 h), and 51.13 ± 4.08% vs. 33.33 ± 1.80% (48 h) (Figure 2E and 2F). Using flow cytometry, erythroid-lineage cells were also enumerated by measuring CD235a, which indicated a similar negative effect of miR-150. Increased expression of miR-150 resulted in fewer CD235a-positive cells during hemin induction relative to each corresponding control sample (Figure 2G and 2H).

**Figure 3:**
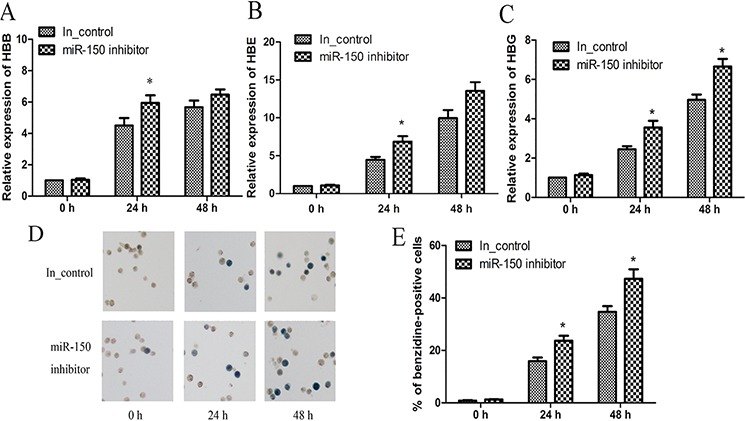
miR-150 inhibitor promotes erythroid differentiation of K562 cells K562 cells were transiently transfected with miR-150 inhibitor or the inhibitor control. At 12 h after transfection, cells were induced were induced by hemin and evaluated at 0, 24 or 48 h after induction. Real-time qRT-PCR analyses of HBB **A.** HBE **B.** and HBG **C.** mRNA levels. Note that GAPDH was used as the control gene and that results were normalized to the control group at 0 h. Representative images **D.** and statistics **E.** of benzidine staining of hemoglobin-containing cells. All data represent mean ± SD (*n* = 3). *P < 0.05 and **P < 0.01 for each paired condition with and without miR-150 inhibitor. In_control = inhibitor control.

In addition to the miR-150 overexpression, miR-150 was inhibited by the transfection of a miR-150 inhibitor and an inhibitor control into K562 cells. In contrast to miR-150 overexpression, miR-150 inhibition resulted in higher expression of HBB, HBG and HBE relative to the inhibitor control group at each time point after hemin induction (Figure 3B and 3C). Benzidine staining again demonstrated that miR-150 inhibition abrogated the negative control of miR-150 in affecting hemin-induced hemoglobin production, as miR-150 inhibitor yielded more benzidine-positive cells compared to each corresponding inhibitor control group (Figure 3D and 3E). Taken together, all of these findings suggest that miR-150 negatively regulates the hemin-induced erythroid differentiation of K562 cells.

### miR-150 inhibits the EPO-dependent differentiation of CD34^+^ cells

**Figure 4:**
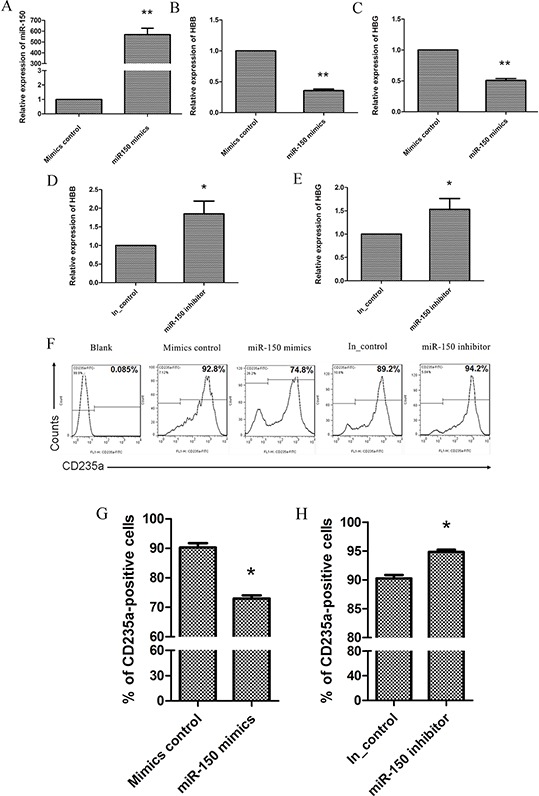
miR-150 suppresses terminal erythroid differentiation of human CD34^+^ cells During EPO-induced CD34^+^ cell erythroid differentiation, cells were transfected on day 6 and day 8 with miR-150 mimic / mimic negative control or miR-150 inhibitor / inhibitor control, and they were then harvested and evaluated on day 10. Real-time qRT-PCR analysis of miR-150 **A.** HBB **B.** and HBG **C.** RNA or mRNA levels in cells transfected with mimic control vs. miR-150 mimic. Real-time qRT-PCR analysis of HBB **D.** and HBG **E.** mRNA expression in cells transfected with miR-150 inhibitor or inhibitor control. Representative images **F.** and Statistics **G.** and **H.** of flow cytometry analysis of CD235a-positive cells. All data represent mean ± SD (*n* = 3), *P < 0.05 and **P < 0.01 for each paired condition; blank = untreated CD34^+^ cells.

To further confirm that miR-150 functions in EPO-induced erythroid differentiation in purified CD34^+^ cells from human cord blood, miR-150 mimics, miR-150 inhibitor, and the corresponding controls were transfected into the erythroid precursors twice, on day 6 and day 8. On day 10, transfected cells were collected and evaluated. Compared to the negative controls for the mimics, transfection of miR-150 mimics significantly increased the functional RNA levels of miR-150 (Figure 4A), and it also suppresses HBB and HBG expression (Figure 4B and 4C). Consistently, the miR-150 inhibitor promotes the expression of HBB and HBG (Figure 4D and 4E). Meanwhile, flow cytometry results demonstrate that transfection of miR-150 mimics produced fewer CD235a-positive cells than did treatment with the mimic controls (73 ± 1.8% vs. 90.3 ± 2.5%, *P* < 0.05), and miR-150 inhibitor correspondingly produced more CD235a-positive cells than the inhibitor control treatment (94.9 ± 0.6% vs. 90.2 ± 1.1%, *P* < 0.05) (Figure 4F and 4H). All of these results confirm the negative role of miR-150 during terminal erythroid differentiation.

### miR-150 suppresses hemin-induced erythroid proliferation in K562 cells

**Figure 5:**
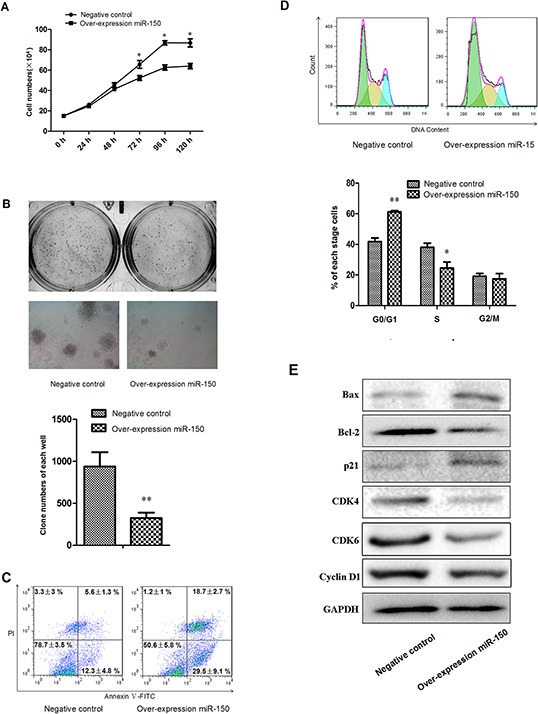
miR-150 suppresses the proliferation of K562 cells **A.** Growth curve of K562 cells stably transfected with pre-miR-150 expression plasmid or negative control plasmid and induced with hemin for 0, 24, 48, 72, 96 and 120 hours. **B.** Representative images and statistics of Soft agar colony formation assay: colony formation was observed and counted in each well of 6-well plate. The left images represent clones formed from control cells, and the right images represent results from cells overexpressing miR-150. **C.** Representative flow cytometry plots with data label of cells assayed with Annexin V and PI. **D.** Representative flow cytometry profiles of cell cycle with statistics shown below. **E.** Western blot analyses of key molecules involved in apoptosis and cell cycle regulation, including Bcl-2, Bax, p21, CDK4, CDK6 and CyclinD1. GAPDH was used as the housekeeping gene. All data represent mean ± SD (*n* = 3). *P < 0.05 for each paired condition with and without miR-150 overexpression.

Because erythroid proliferation is another important aspect of erythropoiesis, we used the stable K562 cell line containing the miR-150 overexpression vector or the control vector to address whether miR-150 modulate erythroid proliferation. Using the CCK-8 cell growth assay, we found that cells overexpressing miR-150 exhibited a delayed growth trend compared to the control groups with presence of hemin induction (Figure 5A). The colony formation experiments produced further evidence of forced miR-150 expression slowing down colony formation by more than 2 folds (*P* < 0.01, Figure 5B). To discover why forced miR-150 caused inhibition of erythroid proliferation, we hypothesized that ectopic miR-150 expression might lead to apoptosis or cell cycle arrest in erythroid cells. Using flow cytometry, we assayed apoptosis with Annexin V and PI double staining, and we found that miR-150 overexpression significantly increased both early (Annexin V^+^/PI^−^, 18.7 ± 2.7% vs. 5.6 ± 1.3%, *P* < 0.05) and late (Annexin V^+^/PI^+^, 29.5 ± 9.1% vs. 12.3 ± 4.8%, *P* < 0.05) apoptosis fractions relative to the control (Figure 5C). Cell cycle detection showed that most cells were blocked at the G0/G1 phase (61.23 ± 0.9%) in erythroid-lineage K562 cells overexpressing miR-150 compared to the control cells (41.80 ± 2.81%) (Figure 5D). Western blot analyses indicated that some important master regulators of apoptosis or cell cycle were altered by forced miR-150 (Figure 5E). Protein levels of Bax and p21 were increased due to miR-150 overexpression relative to the control, whereas those of Bcl-2, CDK4, CDK6 and CyclinD1 were decreased. These alterations are consistent with the functions of these genes, as Bcl-2 is known to be an apoptosis inhibitor, but it interacts with Bax to activate apoptosis [36–39]. p21 has been confirmed to trigger cell cycle arrest at G1, and CDK molecules have the opposite effect [40–43].

### 4.1R is the target gene of miR-150 in terminal erythropoiesis

**Figure 6:**
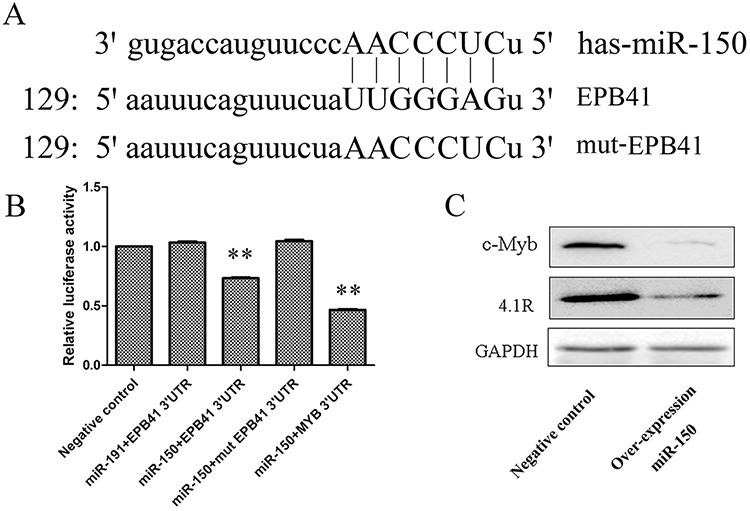
4.1R is a direct target of miR-150 during terminal erythroid differentiation **A.** The highly conserved miR-150-binding motif in the 3′ UTR of 4.1R (EPB41) mRNA predicted from http://www.targetscan.org. The artificially mutated sequence of the 3′ UTR of 4.1R (mut-EPB41) was shown in capital letters. **B.** Relative luciferase activity of the indicated EPB41 reporter construct in 293T cells. Cells were transfected with empty luciferase reporter plasmid, or cotransfected with by EPB41 3′ UTR, mutated EPB41 3′ UTR (mut EPB41 3′ UTR) or MYB 3′ UTR recombinant luciferase reporter plasmid and miR-191 or miR-150 recombinant overexpression plasmid. The luciferase assay was performed at 48 h after transfection. Data was shown as mean ± SD; *n* = 3; **P < 0.01 compared with the negative control. **C.** Western blot for the change in expression of 4.1R and c-Myb at 48 h after hemin induction in stable miR-150-overexpressing and control K562 cell lines.

Unlike the constant inhibition of miR-150 during terminal erythropoiesis, 4.1R (EPB41) protein expression was continued to increase dramatically during terminal erythropoiesis [44–45]. Bioinformatics analysis predicted that the red blood cell membrane protein 4.1R is a direct target of miR-150, and it contains a complete complementary motif with miR-150 at its mRNA 3′ UTR (Figure 6A). To determine whether the predicted target site of 4.1R for miR-150 is a functional target site, we mutated the seed sequence in the 3′ UTR of 4.1R (Figure 6A). A reporter assay using cotransfection with psiCHECK2–4.1R 3′ UTR, psiCHECK2-mut 4.1R 3′ UTR and pSUPER-miR-150 demonstrated that miR-150 can bind to the 3′ UTR of 4.1R mRNA since the cotransfection resulted in a reduction in the activities of firefly luciferase relative to the empty control, which was then abrogated when the 3′ UTR of 4.1R mRNA is mutated (Figure 6B). miR-191 was also used as a negative miRNA control of miR-150 and was shown to have no effect on 4.1 R, as the cotransfection of psiCHECK2–4.1R 3′ UTR and pSUPER-miR-191 did not influence the luciferase activities (Figure 6B). Meanwhile, we used the confirmed miR-150 target MYB [29–30, 46–47] as a positive target control of the reporter assay, and we observed the expected reduction of luciferase activities after cotransduction with psiCHECK2-MYB 3′ UTR and pSUPER-miR-150 (Figure 6B). Western blotting further confirmed the above observation (Figure 6C). The protein levels of 4.1R were attenuated in K562 cells overexpressing miR-150 relative to control cells.

### miR-150 overexpression results in changes to gene profiles that are associated with the regulation of cell differentiation and proliferation

**Figure 7:**
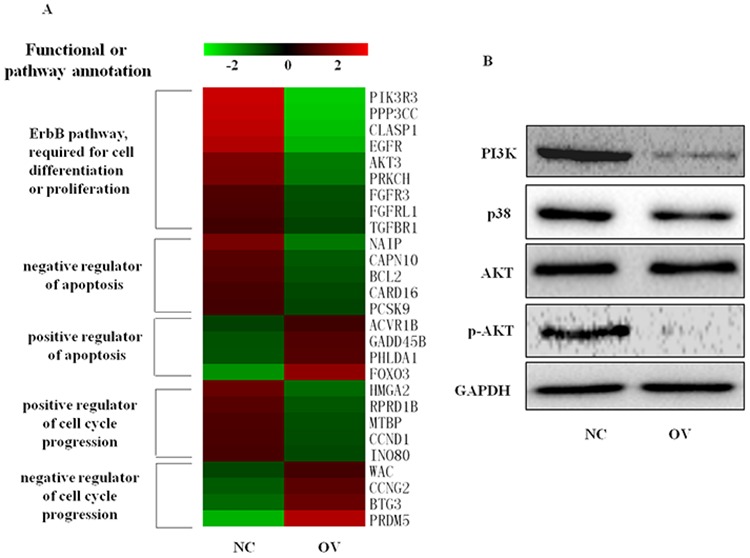
Overexpression of miR-150 changes gene expression profiles, including cell cycle regulators and genes involved in ErbB signaling and apoptosis **A.** Heat map of representative genes differentially expressed in erythroid cells derived from hemin-induced K562 cells with (OV) or without (NC) miR-150 overexpression, detected by GeneChip^®^ Human Genome U133 Plus 2.0 Array, Affymetrix (Santa Clara, CA, USA). Note: Heat map was generated using MEV. Genes were annotated based on http://david.abcc.ncifcrf.gov and http://www.genecards.org/. **B.** Western blot for the changes in MAPK-p38 or PI3K-AKT signaling at 48 h after hemin induction in stable miR-150-overexpressing and control K562 cell lines.

Although 4.1R is shown to be a direct target of miR-150, the erythroid 4.1R knockout mice did not show disorders of early-stage erythropoiesis [48], and siRNA-mediated 4.1R depletion did not affect terminal erythropoiesis (data not shown). MYB is a well known target of miR-150 affecting multiple hematopoietic lineages, while we found that endogenous MYB protein was not increased with the decrease of miR-150 through terminal erythropoiesis in EPO-induced CD34^+^ cells (Supplementary Figure S1). We therefore concluded that the other related genes and pathways involved in regulation of erythroid differentiation and proliferation resulted from ectopic miR-150 expression. Using the latest version of the GeneChip^®^ Human Genome U133 Plus 2.0 Array transcription chip, 1019 genes were found to be suppressed in the miR-150-overexpressing K562 cells relative to the control group, and the expression levels of another 1045 genes were enhanced by miR-150 overexpression. Gene expression profiling was further verified by qRT-PCR assay of 8 representative down-regulated or up-regulated genes (Supplementary Figure S2). These differently expressed genes function differently, including 47 genes for regulators of cell differentiation (*P* = 0.002) and 67 genes for regulators of cell proliferation (*P* = 0.003), mainly included in the ErbB-MAPK-P38 or ErbB-PI3K-AKT signaling pathways (Figure 7A), which were confirmed by Western blot (Figure 7B). Overexpression of miR-150 reciprocally increased expression of genes responsible for apoptosis activation and cell cycle arrest and suppressed expression of genes involved in apoptosis inhibition and cell cycle progression (Figure 7A). For example, compared to controls, miR-150 overexpressing cells showed decreased mRNA levels of Bcl-2 and NAIP, which have previously been described as apoptosis inhibitors [37–38, 49], and elevated levels of Foxo3 and PHLDA1, which are thought to be apoptosis triggers [50–53]. Similarly, exogenous expression of miR-150 up-regulated PRDM5 and BTG3, which have been reported to cause cell cycle arrest and apoptosis [54–57], and down-regulated CCND1 and MTBP, which function to activate cell cycle progression [58–61]. These effects of miR-150 on the regulation of erythroid cell apoptosis and cell cycle progression were confirmed, shown in Figure 5.

Differentially expressed genes were further analyzed for the possibility of being miR-150 direct target genes. Top fifty genes were predicted as direct target genes of miR-150, and most of them were showed interesting annotations such as actin-related, cellular compartment, and cytoskeleton organization, including SORBS3, NF2, ATM, ARHGEF10L, and CXCL12 according to functional annotations of DAVID Bioinformatics Resources 6.7 (http://david.abcc.ncifcrf.gov). 4.1R was excluded from these top fifty target genes as miR-150 overexpression did not result in significant decline of 4.1R mRNA, suggesting that miR-150 could suppress translation of 4.1R directly.

## DISCUSSION

During erythropoiesis, a spatiotemporally distinct set of miRNAs is thought to play an important role [22, 62–65]. For instance, weak expression of miR-150 and miR-222 coupled with abundant expression of miR-451, miR-144 and miR-146b has been described in human erythrocytes from EPO-induced CD34^+^ cells and adult bone marrow [25, 28–29]. In our study, we first assessed the expression profile of miR-150 during terminal erythroid differentiation in EPO-induced cord blood CD34^+^ cells or hemin-induced K562 cells, and we observed a constant decline in miR-150 expression. Based on the current research, we propose a hypothesis in which miR-150 is a negative regulator of terminal erythroid differentiation.

To test this hypothesis, human K562 erythroleukemia cells, which have been proven to be a very useful tool for erythroid-lineage development research, were induced into erythroid differentiation with 50 μM hemin [28, 34–35, 66–67]. As expected, forced expression of miR-150 suppressed hemin-dependent erythropoiesis in K562 cells; commitment to hemoglobinization and GPA marking were reduced compared to the controls at each time point. Coincidence, in the loss-of-function experiments, K562 cells transfected with miR-150 inhibitor showed increased erythroid differentiation, as the specific inhibitor induced the expression of hemoglobin genes and the appearance of the erythrocyte membrane protein GPA relative to the mock-inhibited cells. We also performed gain- and loss-of-function experiments with miR-150 at the late phase of EPO-induced erythropoiesis in human CD34^+^ cells, and these results validated the above observations. miR-150 mimics dramatically reduced the expression of hemoglobin genes, while miR-150 inhibitor induced hemoglobinization and the presence of CD235a in EPO-induced erythrocytes on day 10. Surprisingly, forced expression of miR-150 was also shown to restrain erythroid proliferation via inducing apoptosis and cell cycle arrest. Both Western blot and genome-wide expression chip assays confirmed that expression of key molecules involved in apoptosis and cell cycle were altered due to the artificially overexpressed miR-150. These findings confirm that constant down-regulation of miR-150 is essential to ensure successful terminal erythropoiesis. In a recent report, Kouhkan et al. found that the administration of anti-miR-150 was useful for driving megakaryocyte-erythrocyte progenitor differentiation into the erythroid lineage in the absence of growth factors and cytokines [68].

Along with the ongoing down-regulation of miR-150, the red cell membrane protein 4.1R (EPB41) was up-regulated during terminal erythropoiesis [44–45]. Both bioinformatics prediction and the luciferase reporter assay indicated that 4.1R is a target gene of miR-150. miR-150 overexpression reduced the protein level of 4.1R. Erythrocyte membrane formation is crucial to erythrocytes and is involved in erythroid proliferation and differentiation because it enables erythrocytes to circulate in capillaries. 4.1R knockout mice showed that loss of 4.1R compromises membrane skeleton assembly in erythroid progenitors [48, 69–70]. The genome-wide miRNA expression profiling assay via GeneChip^®^ Human Genome U133 Plus 2.0 Array also indicated a set of possible miR-150 target genes in K562 cells, including SORBS3, NF2, ATM, ARHGEF10L, and CXCL12. Interestingly, these genes are involved in actin-related, cellular compartment, and cytoskeleton organization categories based on GO annotation (http://david.abcc.ncifcrf.gov/). Because each miRNA can target multiple mRNAs, we predict that miR-150 might affect the erythroid lineage via modulating different cellular structure protein-coding genes at different stages. The regulatory mechanism for forming the unique erythrocyte membrane skeleton has not been well studied. This report is the first to indicate the interaction between miRNAs and erythrocyte membrane proteins, which in turn will broaden our understanding of the functions of miRNAs during erythropoiesis.

4.1R was found to be a new target of miR-150, but knockdown of 4.1R did not affect terminal erythropoiesis, and the ratio of nucleated erythroblasts in the 4.1R knockout mouse was normal [48]. The known target of miR-150, MYB [29–30, 46–47], has been reported to promote the early erythropoiesis [71–73]. But MYB is not increased during terminal erythropoiesis in EPO-induced human CD34^+^ cells, the evidence of miR-150 targeting MYB in human physiological terminal erythroblast differentiation is not sufficient. A transcriptional profiling assay provided global insight into the alteration of gene crosstalk introduced by forced miR-150 during terminal erythropoiesis in hemin-induced K562 cells. Here, we found that aberrantly expressed miR-150 suppressed the two major signaling pathways involved in erythroblast proliferation and survival, ErbB-MAPK-p38 and ErbB-PI3K-AKT. Activation of the p38 MAPK pathway has been reported to be a prerequisite for inducing erythroid differentiation of K562 cells [74–75]. TfR1 engagement increased cell sensitivity to EPO to induce erythropoiesis via activating PI3K-AKT pathways [76]. PI3K/AKT signaling is also essential for cell proliferation via inactivating several downstream molecules, such as Foxo3, to inhibit cell growth [51]. Our results indicate that miR-150 overexpression clearly suppressed activation of these two pathways during terminal erythropoiesis, thus explaining the negative effect of miR-150 on erythropoiesis.

In conclusion, constant miR-150 suppression is essential for normal terminal erythroid development, but more research is needed to elucidate upstream regulators, the mechanism underlying the effect of miR-150, and especially the correlation between aberrant miR-150 expression and anemic disorders in the clinic, which are rarely studied.

## MATERIALS AND METHODS

### Purification and culture of human CD34^+^ cells

CD34^+^ cells were purified from human umbilical cord blood (UCB, provided from normal full-term deliveries after informed consent as approved by the Maternal and Child Health Hospital of Hunan Province, Hunan, China) by positive selection using the CD34^+^ magnetic selective beads system (Miltenyi Biotec, Germany) according to the manufacturer's instructions. The purification of CD34^+^ cells were checked by CD34 antibody (BD) staining and flow cytometry analysis. Cells were first cultured at 10^5^ cells/ml for 6 days in Serum Free Expansion Medium (SFEM, Stem Cell Technologies) supplemented with 10% fetal bovine serum (FBS, Stem Cell Technologies), 10 ng/ml stem cell factor (SCF), 1 ng/ml IL-3, and 1 IU/ml erythropoietin (Stem Cell Technologies) at 37°C in 5% CO_2_, and then cultured for 8 more days in the above complete medium with the presence of 30% FBS and the absence of IL-3 and SCF.

### Cell culture

K562 cells (ATCC CCL-243) were obtained from the American Type Culture Collection (ATCC, USA). K562 cells were grown in RPMI 1640 media (Gibco, USA) supplemented with 10% FBS (Gibco, USA) at 37°C in 5% CO_2_. Hemin (50 μM, Sigma, USA) was used to induce erythroid differentiation of K562 cells. The growth assay was carried out using Cell Counting Kit-8 (CCK-8) kit (Dojindo, Japan). HEK293 cells (ATCC CRL-1573) were grown in DMEM (Gibco, USA) with 10% FBS (Gibco, USA) at 37°C in 5% CO_2_.

### Oligonucleotides, plasmid construct and cells transduction

The miR-150 mimic, mimic control, inhibitor and inhibitor control were all obtained from Dharmacon. K562 cells were transiently transduced with these oligonucleotides using DharmFECT1 (Dharmacon, USA) at a final concentration of 100 nM. At 12 hours after transfection, cells were induced by hemin and evaluated at 0, 24, 36 or 48 hours after induction. For human CD34^+^ cells, the miR-150 mimic, mimic control, miR-150 inhibitor or inhibitor control was transduced into cells under the same conditions as above on culture day 6 and repeated one more time on day 8, and the cells were then harvested and assessed on day 10. Recombinant overexpression plasmids for miR-150 and miR-191, including pSUPER-miR-150 and pSUPER-miR-191, were obtained from OligoEngine. K562 cells were first transfected with the miR-150 overexpression vector, pSUPER-miR-150, and then selected by puromycin at the concentration of 1 μg/mL until a stable cell line was generated, and it was then sustained in 200 ng/ml puromycin. 3′ UTRs of EPB41 (4.1R), mutated 3′ UTRs of EPB41 and MYB (c-Myb) were first amplified using PCR primers (Table 1) and then cloned into the psiCHECK2 reporter plasmid (Promega, USA).

**Table 1:** Human specific primers used for PCR

Gene	Forward primer (5′ -> 3′)	Reverse primer (5′ -> 3′)
Pre-mir-150	GAAGATCTTCTACTTTGCGCATCA CACAGA	CCGCTCGAGCGGCCCTTGCTGGTTCTCTACTG
HBB	CTGCTGGTGGTCTACCCTTG	TGGACAGCAAGAAAGCGAGC
HBG	GAGAAACCCTGGGAAGGCTC	TGTGCCTTGACTTTGGGGTT
HBE	CAGAGAGGCAGCAGCACATA	TGCACTTCAGGGGTGAACTC
GAPDH	CATGAGAAGTATGACAACAGCCT	AGTCCTTCCACGATACCAAAGT
EPB41 3′UTR	ccgCTCGAGCCAACTCTGCCCTTCT CCCAT	ggGTTTAAACAAACCACCCGCAACAAAGGA
MYB 3′ UTR	ccgCTCGAG GACATTTCCAGAAAA GCAT	ggGTTTAAAC AGGTAAAATAAGGGCACATCT
mut EPB41 3′UTR	AGTTTCTACCcccGATTTA TACCAAGAGATTCTTCTAGATC	TAAATCgggGGTAGAAACTGAAATTAATTTTCTGGTG

### RNA extraction and quantitative real-time RT-PCR

Total RNA was isolated using TRIzol reagent (Invitrogen, USA). cDNA was synthesized by Revert Aid First Strand cDNA Synthesis Kit (Thermo Scientific, USA). The RT reaction for miR-150 was carried out with stem-loop RT primers (RiboBio, China). Quantitative PCR was performed using SYBR Green qPCR Master Mixes (Takara, China). Relative expression was determined using U6 (primers from RiboBio, China) as the internal control for miRNAs and GAPDH as the internal control for mRNAs of other genes (primers listed in Table 1 and Supplementary Table S1).

### Benzidine staining

Benzidine dihydrochloride (Sigma, USA) solution was prepared with 0.5 M ethylic acid. Immediately before use, 1 μl 30% hydrogen peroxide was added to 50 μl benzidine solution. Then, 1 μl fresh whole benzidine solution containing hydrogen peroxide was added in 10 μl K562 cells. Benzidine-positive cells were stained blue, whereas benzidine-negative cells were light yellow.

### Western blot analyses

Whole-cell lysates of cultured cells were prepared with RIPA buffer (Thermo Fisher, USA) in the presence of protease inhibitor or PhosStop cocktail (Roche, France). Protein concentration was measured using a Pierce^®^ BCA protein assay kit (Thermo Fisher, USA). Western blot analysis was performed as previously described, and probed using primary antibodies anti 4.1R (kindly offered by Red Cell Physiology Laboratory, New York Blood Center, NY, USA. Hu 2013), c-myb, p21, Bax, Bcl-2, CDK4, CDK6, CyclinD1, PI3K, p38, p-AKT, AKT, GAPDH and HRP conjugated secondary antibodies (Santa Cruz).

### Flow cytometry analyses

All the reactions were performed under conditions of antibody saturation. Unstained cells were used as a negative control. Flow cytometry analyses were finished within 1 h after staining. To analyze the cell-surface protein CD235a, 2 × 10^5^ cells were suspended in 100 μl PBS supplemented with 0.5% BSA and then stained with fluorochrome-conjugated antibodies (CD235a-PE, BD Pharmingen, USA) for 30 min on ice. Cells were washed twice with PBS-0.5% BSA before analysis. For cell cycle analysis, 1 × 10^6^ cells were washed in PBS twice, incubated in 10 μl membrane permeating solution for 1 min on ice, then stained with 100 μl PI for 20 min on ice (in the dark) and re-suspended with 300 μl PBS prior to FACS analysis (Beckman Coulter, USA). Apoptosis analysis was carried out using an Annexin V-FITC/PI double staining kit (Miltenyi Biotec, Germany). After twice PBS washing, 1 × 10^6^ cells were first stained with Annexin V-FITC for 10 min, then stained with PI for 20 min, and kept on ice prior to FACS analysis. All data analysis was performed using the FlowJo 7.5.5 suite of flow cytometry analysis tools.

### Soft agar colony formation assay

The bottom layer of a 6-well plate was prepared by pouring 3 mL of Basal Medium Eagle (Sigma, USA) containing 0.5% agar (Becton, Dickinson and Company, USA), 10% serum, 100 μg/ml gentamicin and 200 μg/ml glutamine into each well, and then allowed to solidify. K562 cells at 8 × 10^3^ cells/ml were re-suspended in medium containing 0.3% agar, 10% serum, 100 μg/ml gentamicin and 200 μg/ml glutamine; 1 mL of this solution was poured as a top layer in each well. The cells were incubated for 15 days at 37°C and 5% CO_2_ prior to colony counting.

### Luciferase reporter assay

293T cells were plated into 24-well plates at 1.5 × 10^5^ cells/well 24 h before transfection. Then, 30 ng/well recombinant reporter plasmid (psi-EPB41 3′ UTR, psi-mutated EPB41 3′ UTR or psi-MYB 3′ UTR) and recombinant pSUPER-miRNA plasmid expressing miR-150 or miR-191 were co-transfected into each well by using the Attractene transfection reagent (QIAGEN, USA) in triplicate. Luciferase assays were performed 24 h after transfection by using the Dual-Luciferase Reporter Assay System (Promega, USA) on a Berthold AutoLumat LB953 rack luminometer.

### Transcriptional profiling and data analysis

Total RNA was extracted and purified from K562 cells stably overexpressing miR-150 and control K562 cells using TRIzol and RNeasy Kit (QIAGEN, USA). cDNAs were prepared and analyzed using GeneChip^®^ Human Genome U133 Plus 2.0 Array, Affymetrix (Santa Clara, USA) as previously described [77] with technical support from Shanghai Biotechnology Corporation. Direct target genes of miR-150 in present study were selected based on more than 2-fold of reduction together with array detect signal more than 5 in both samples. Genes showing fold changes greater than 2 and array-detected signals greater than 7 in at least one sample were selected as differently expressed genes.

### Bioinformatics analysis

All target genes of miR-150 were predicted by both TargetScan Human 6.2 (http://www.targetscan.org) and Human MicroRNA Targets Database (http://www.microRNA.org), and selected based on a prediction score greater than 0.5. The DAVID Functional Annotation Tool (DAVID Bioinformatics Resources 6.7) was used to annotate GO terms and KEGG pathways for differently expressed genes found in CHIP (http://david.abcc.ncifcrf.gov), with GeneCard (http://www.genecards.org/) used as a supplementary reference. The heat map of differently expressed genes was drawn using MultiExperiment Viewer 4.6 (The Institute for Genomic Research, US) software.

### Statistical analysis

All data are indicated as mean ± SD. All results were analyzed using SPSS 15.0 software. Significant differences between the groups were determined by analysis of variance and Tukey's range test. Differences among groups were considered statistically significant at *p* < 0.05.

## SUPPLEMENTARY FIGURES AND TABLE


