# Supplementary material for: miR-150 inhibits terminal erythroid proliferation and differentiation
Source: Oncotarget. 2015 Nov 3;6(40):43033–47. doi: 10.18632/oncotarget.5824 (PMC4767489; doi:10.18632/oncotarget.5824)
Supplement: Supplementary file 1 [file oncotarget-06-43033-s001.pdf]

SUPPLEMENTARY FIGURES AND TABLE

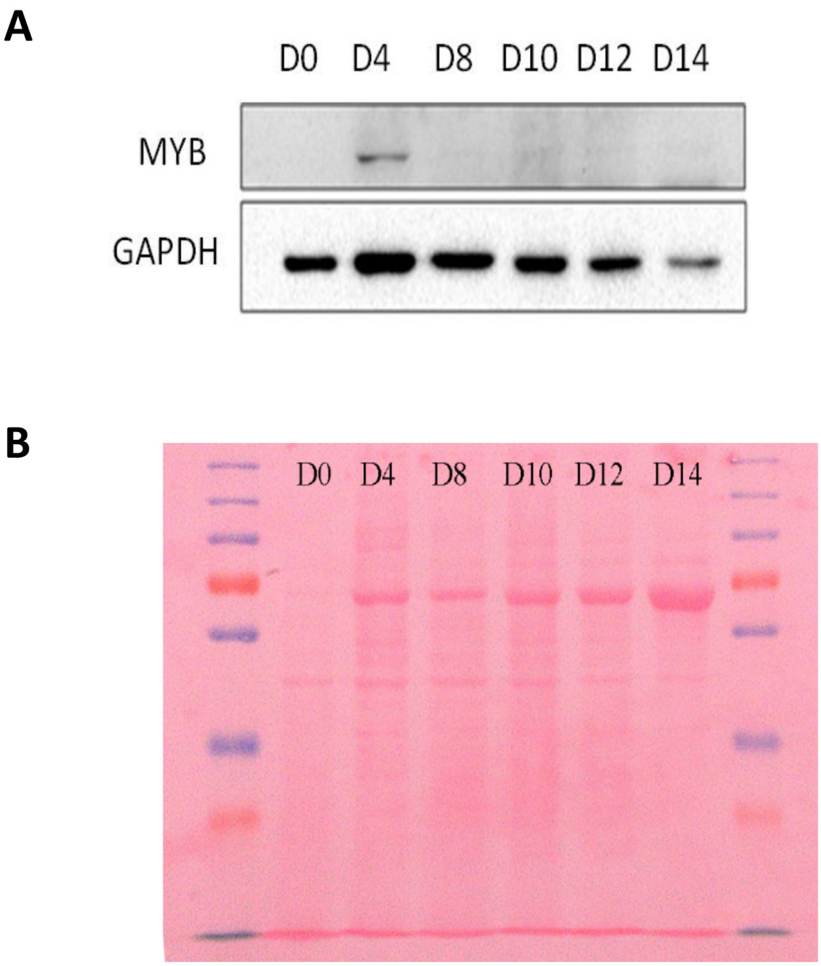

Supplementary Figure S1: Changes of MYB protein level during EPO-induced CD34⁺ cells. A. Western blot analysis of MYB on culture days 0, 4, 8, 10, 12 and 14. **B.** The same membrane from the above assay stained by Ponceau S.

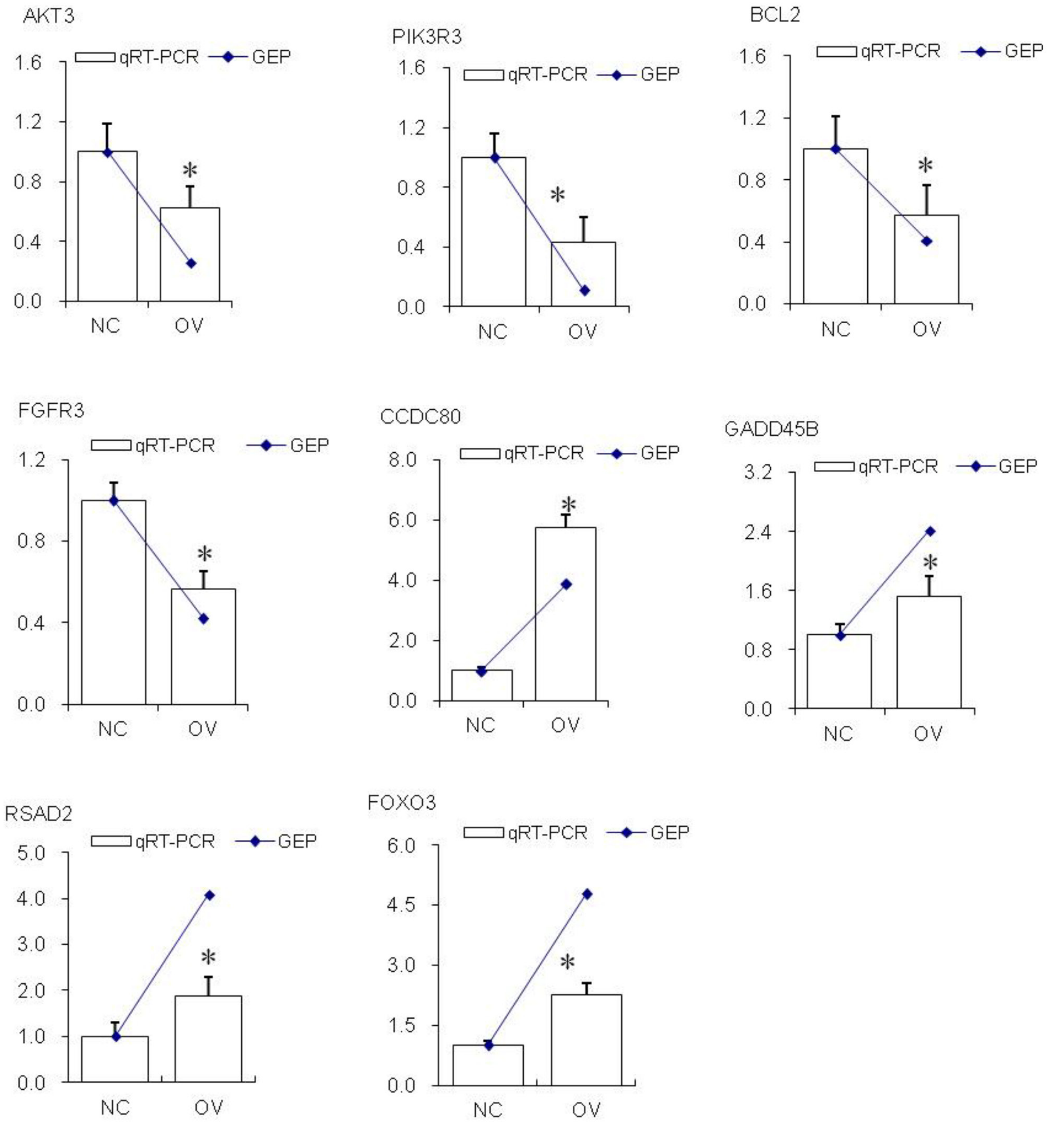

Supplementary Figure S2: qRT-PCR verification of genes expression profiling (GEP) in erythroid cells derived from hemin-induced K562 cells with (OV) or without (NC) miR-150 overexpression. All data were normalized to the NC treatment. Values shown are the mean± SEM ($n = 3$). *$P < 0.05$ compared with the negative control.

Supplementary Table S1: Human specific primers of qRT-PCR for verifying transcriptional profiling

Gene	Forward primer (5′- > 3′)	Reverse primer (5′- > 3′)
RSAD2	CGTGAGCATCGTGAGCAATG	TCCCTACACCACCTCCTCAG
CCDC80	CAATTTTGGTCTGCGCCACA	ATGGAGAAGTACTCCGGGCT
PIK3R3	AAAGTCGAGATGGAGAGCAGC	AGTCATTGGCTTAGGTGGCT
FOXO3	TGTTGGTTTGAACGTGGGGA	TGTCCACTTGCTGAGAGCAG
AKT3	GAGGGGAGTCATCATGAGCG	ACTGGCATTTTGCCACTGAA
FGFR3	GGAGTTCCACTGCAAGGTGT	TCCTTGTCGGTGGTGTTAGC
BCL2	GAACTGGGGGAGGATTGTGG	CCGTACAGTTCCACAAAGGC
GADD45	CGTTGGTTTCCGCAACTTCC	AAAATCCGAGCCAGAGAGCC
